# Comparison of targeted next-generation sequencing and metagenomic next-generation sequencing in the identification of pathogens in pneumonia after congenital heart surgery: a comparative diagnostic accuracy study

**DOI:** 10.1186/s13052-024-01749-z

**Published:** 2024-09-12

**Authors:** Yi-Rong Zheng, Xiu-Hua Chen, Qiang Chen, Hua Cao

**Affiliations:** 1 Department of Cardiac Surgery, College of Clinical Medicine for Obstetrics & Gynecology and Pediatrics, Fujian Medical University, Fujian Children’s Hospital (Fujian Branch of Shanghai Children’s Medical Center), Fuzhou, China; 2 College of Clinical Medicine for Obstetrics & Gynecology and Pediatrics, Fujian Medical University, Fujian Children’s Hospital (Fujian Branch of Shanghai Children’s Medical Center), Fuzhou, China

**Keywords:** Targeted next-generation sequencing, Metagenomic next-generation sequencing, Pneumonia, Infection, Congenital heart disease

## Abstract

**Background:**

This study aimed to compare targeted next-generation sequencing (tNGS) with metagenomic next-generation sequencing (mNGS) for pathogen detection in infants with severe postoperative pneumonia after congenital heart surgery.

**Methods:**

We conducted a retrospective observational study using data from the electronic medical record system of infants who developed severe pneumonia after surgery for congenital heart disease from August 2021 to August 2022. Infants were divided into tNGS and mNGS groups based on the pathogen detection methods. The primary outcome was the efficiency of pathogen detection, and the secondary outcomes were the timeliness and cost of each method.

**Results:**

In the study, 91 infants were included, with tNGS detecting pathogens in 84.6% (77/91) and mNGS in 81.3% (74/91) of cases (*P* = 0.55). No significant differences were found in sensitivity, specificity, PPA, and NPA between the two methods (*P* > 0.05). tNGS identified five strains with resistance genes, while mNGS detected one strain. Furthermore, tNGS had a faster detection time (12 vs. 24 h) and lower cost ($150 vs. $500) compared to mNGS.

**Conclusion:**

tNGS offers similar sensitivity to mNGS but with greater efficiency and cost-effectiveness, making it a promising approach for respiratory pathogen detection.

**Supplementary Information:**

The online version contains supplementary material available at 10.1186/s13052-024-01749-z.

## Introduction

Pneumonia is one of the important causes of postoperative morbidity and mortality in children with congenital heart disease (CHD). The risk of postoperative infection in these children is significantly increased by frequent combinations of malnutrition, pulmonary congestion, ischaemia–reperfusion injury after cardiopulmonary bypass, and congenital immunodeficiency [1]. Early identification of pathogens and targeted anti-infective treatment is key to the treatment of pulmonary infections while helping reduce problems of antibiotic misuse and resistance [2]. In recent years, metagenomic next-generation sequencing (mNGS) of bronchoalveolar lavage fluid (BALF) has been widely used in clinical practice. Studies have shown that mNGS can sequence as much DNA and/or RNA as possible in a sample, can detect rare or unknown pathogens, help guide the rational use of antibiotics, shorten the duration of mechanical ventilation, and improve the prognosis of patients with pulmonary infections [3–7]. However, mNGS is expensive, greatly influenced by human genes, and has a long detection time. Thus, target next-generation sequencing (tNGS), a more rapid and economical technique, was employed. It can enrich specific pathogen genomes from clinical samples and selectively sequence genes of interest, which may increase detection sensitivity, improve timeliness and costs and enable early diagnosis of respiratory pathogens [8]. However, only a few studies have directly evaluated the performance of mNGS and tNGS in the diagnosis of pulmonary infection pathogens. Therefore, we tried to evaluate the clinical application value of tNGS by detecting BALF pathogens in patients with severe pneumonia after congenital heart surgery (CHS).

## Methods

### Patients and study criteria

We conducted a retrospective cohort study of the data of infants from the electronic medical record system of Fujian Children’s Hospital. The study population was infants with postoperative severe pulmonary infection who underwent CHS between May 2021 and May 2022. The diagnosis of pneumonia was based on the criteria set by the American Academy of Paediatric Infectious Diseases and the Infectious Diseases Society of America [9]. Demographic, clinical, laboratory, treatment, and outcome data were extracted from the electronic medical records. Infants with severe pulmonary infections after CHS and whose BALF was sent for tNGS or mNGS tests were included in the study. By contrast, infants who had contaminated BALF samples by leakage, immunodeficiency, and incomplete medical history were excluded.

This study was approved by the ethics committee of Fujian Children’s Hospital. Due to the retrospective nature of the study, the need for informed consent was waived by Fujian Children’s Hospital ethics committee. The study was reported in adherence to the Standards for Reporting Diagnostic Accuracy Studies (STARD) guidelines [10]. 

### Sample collection and processing

After written informed consent was obtained from the patients, the attending physician performed a bedside bronchoscopy to collect BALF samples. Each sample was placed in a sterile sputum container and divided into three parts: one part was sent for tNGS examination, the second part for mNGS examination, and the third part for conventional microbiological test (CMT), including gram-stained smear and bacteriological culture, direct immunofluorescence antigen detection, conventional reverse-transcription polymerase chain reaction (RT-PCR) and real-time quantitative PCR (qPCR) in the clinical laboratory. The BALF was stored at − 20 to − 80 °C awaiting use.

### tNGS Procedure and limitations

#### Sample Preparation

BALF samples were centrifuged at 12,000 rpm for 5 min and then ground on a grinder (Tiss-24, Jingxin, Shanghai, China) at 70 Hz for 5 min. The ground samples were then used for DNA/RNA extraction and purification (Zymo BIOMICS DNA Miniprep Kit R2002) according to the manufacturer’s instructions. Extracted DNA was used for library construction.

#### Library Construction

The library was constructed using a multiplex PCR capture library construction kit (Guangzhou Jinqirui Biotechnology Co. Ltd., Guangzhou, China). A set of nucleic acid standards of known concentration was added to the amplification system. During this process, two rounds of PCR were performed. The nucleic acid and cDNA of the sample were used as templates and 306 microorganism-specific primers were selected for multiplex PCR amplification to enrich the target pathogen sequences. Specific primers (at least two primers for each pathogen) were designed to target highly conserved regions of pathogens including 65 bacteria, 14 fungi, 68 viruses, and 6 mycoplasma/chlamydia and 15 drug-resistance genes (Guangzhou Jinqirui Biotechnology Co. Ltd., Guangzhou, China) (Supplementary Tables 1 and Supplementary Table 2). Thereafter, the enrichment of the target region was achieved by PCR to form short fragments of nucleic acid that do not need to be interrupted. After the amplification step, PCR products were bead purified and then amplified using primers with sequencing adapters and different barcodes. After the final amplification product was purified by agarose gel electrophoresis, the quality was detected and quantified with a Qubit 4.0 fluorometer. Under normal circumstances, the 250–350 bp range in the library accounts for no less than 20% and the library concentration is ≥ 0.5 ng/µL.

#### Sequencing

The denatured library was sequenced on a KM MiniSeqDx-CN platform (Illumina Inc., San Diego, CA, USA) using a MiniSeq Rapid Kit (sequencing read length = SE100). Data generated by the KM MiniSeqDx-CN platform were identified and counted by adapters and low-quality reads or reads with lengths of < 50 bp were filtered. Reads with correct alignment were retained and compared with the pathogen database to confirm the pathogen type.

### Limitations and biases of tNGS

#### Target Restriction

tNGS focuses on pre-defined pathogen targets, which means it cannot detect microorganisms outside the targeted panel. This limits its ability to identify rare or emerging pathogens not included in the panel.

#### PCR Bias

The amplification process in tNGS can introduce biases, potentially affecting the relative abundance of detected pathogens.

#### Data interpretation

Interpretation of tNGS results can be arbitrary and is subject to established criteria, which may not cover all clinical scenarios.

### mNGS Procedure and limitations

#### Sample Preparation

Standard procedures were followed to collect 3 mL of BALF from infants. A 1.5-mL microcentrifuge tube was loaded with 0.5 mL of the collected BALF sample and 1 g of 0.5-mm diameter glass beads. This tube was secured to the horizontal attachment of a vortex mixer and vigorously agitated at 3000 revolutions per minute for about 30 min. After vortexing, 0.3 mL of the lysed sample was transferred to a new 1.5-mL microcentrifuge tube for DNA extraction using the TIANamp Micro DNA Isolation Kit (Tiangen Biotechnology) per the manufacturer’s recommendations [4]. Thereafter, DNA libraries were constructed by methods such as DNA fragmentation, end repair, adapter ligation, and PCR amplification.

#### Library Construction

DNA libraries were constructed by DNA fragmentation, end repair, adapter ligation, and PCR amplification. Agilent 2100 was used for DNA library quality control. Qualified libraries were sequenced by the BGISEQ-50 platform [11].

#### Sequencing

**Fig. 1:**
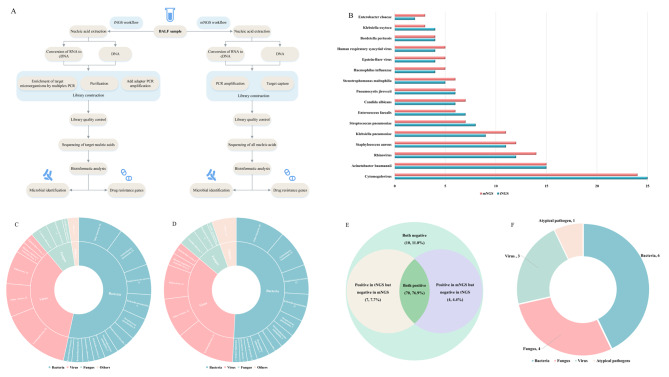
(**A**). Workflow for tNGS and Mngs; (**B**). Distribution of the top 16 microorganisms determined by tNGS and mNGS; Microbial categories of pulmonary infections in infants identified by tNGS (**C**) and mNGS (**D**); (**E**) Shows the positive ratio of tNGS to mNGS; (**F**) Shows microbial categories identified by mNGS but not tNGS

Quality control of raw-sequencing data, including the removal of low-quality, low-complexity, short fragments (< 35 bp) and splice junctions, followed by Burrows–Wheeler alignment to the human reference genome (hg38), was performed, and fragments obtained in the human genome were discarded. The microbial classification was performed by mapping the remaining sequenced fragments to archaeal, bacterial, fungal, protozoan, viral, and parasite genomes in the NCBI genome database [12]. The number of uniquely aligned reads was counted and normalized to obtain the number of reads strictly mapped to the pathogen species and the number of reads strictly mapped to the pathogen genera. The workflow and timeline for tNGS and mNGS are shown in Fig. 1A.

### Limitations and biases of mNGS

#### Human DNA interference

mNGS sequences all DNA in a sample, including human DNA, which can dominate the sequencing reads and reduce the proportion of pathogen sequences, potentially lowering sensitivity for pathogen detection.

#### Cost and Turnaround Time

mNGS is more expensive and has a longer turnaround time compared to tNGS, making it less feasible for rapid and cost-effective diagnosis.

#### Detection sensitivity

Despite its broad target range, mNGS may have lower sensitivity for certain pathogens due to the overwhelming presence of human DNA and other non-target sequences.

### Statistical analysis

Data were analyzed using SPSS software version 25.0 for Windows (IBM Corp., Armonk, NY, USA). Independent continuous variables were presented as the mean ± standard deviation and were analyzed by t-tests. Counts and percentages describe the enumeration data. Means were compared using Student’s t-test and Fisher’s exact test was used for categorical data. The Mann–Whitney U test was applied for non-normally distributed data. A two-sided *P*-value of < 0.05 was regarded as statistically significant. Additionally, the overall agreement and Cohen kappa were assessed by comparing the tNGS group with the mNGS group. Cohen kappa is the standard agreement coefficient, which considers the probability that an agreement will occur by chance. A value of 1 would indicate total agreement between the tests, whereas a value of 0 would mean no agreement.

## Results

### Demographic characteristics

**Fig. 2:**
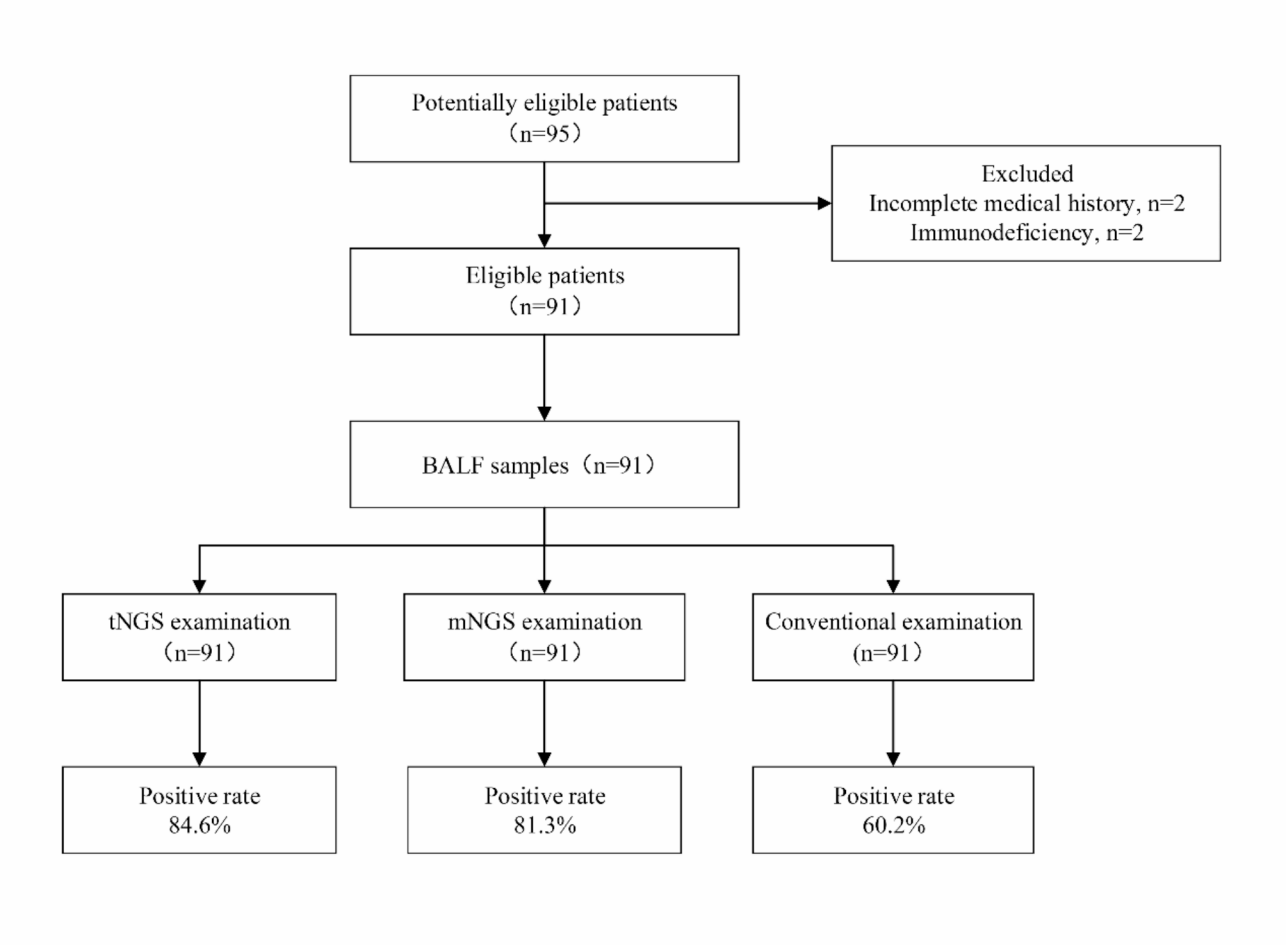
Participant flowchart. BALF, bronchoalveolar lavage fluid

A total of 95 infants with severe pneumonia after CHS were screened in this study, and four infants were excluded according to the exclusion criteria. Among these infants, two had incomplete clinical history data and two had immunodeficiencies. Finally, 91 cases were included in the study (Fig. 2). The baseline characteristics of the tNGS and mNGS groups, including demographic characteristics, laboratory data, and ventilator parameters are shown in Tables 1 and 2, with no statistical significance (*P* > 0.05).

**Table 1:** Clinical characteristics of included patients^a^

Characteristics	Patients (*n* = 91)
Sex (Male/Female)	51/40
Age (months; mean ± SD)	5.5 ± 1.1
Weight (kg; mean ± SD)	5.5 ± 0.8
Pulmonary hypertension, n (%)	59 (65)
Degree of pulmonary hypertension Mild Moderate	
	20 (22%)
	30 (33%)
Severe	9 (10%)
Preoperative respiratory infection, n (%)	43 (47)
Congenital heart disease, n	
VSD	37
VSD + ASD	20
TAPVC	11
COA	7
TOF	6
DORV	5
PDA	5
CPB time (minutes; mean ± SD)	113.7 ± 26.3
Aortic cross-clamp time (minutes; mean ± SD)	55.1 ± 18.8
Postoperative bronchoscopy time (days; mean ± SD)	2.5 ± 0.9

Abbreviations: VSD, ventricular septal defect; ASD, atrial septal defect; TAPVC, total anomalous pulmonary venous connection, COA, coarctation of the aorta; TOF, tetralogy of Fallot; DORV, double outlet of right ventricle; PDA, Patent ductus arteriosus; CPB, cardiopulmonary bypass

^a^Data reported as number and percentage or mean ± standard deviation

**Table 2:** Laboratory data and ventilator parameters during the study period

Variable	Patients (*n* = 91)
WBC (10^9^/L)	12.6 (6.8, 18.8)
Percentage of neutrophils (%)	69.5 (60.1, 81.5)
Hb (g/dL)	11.2 (9.8–13.5)
PLT (10^9^/L)	210 (155–420)
CRP (mg/L)	25.7 (15.6, 52.8)
PCT (ng/mL)	7.0 (2.9, 15.8)
Scr (mmol/L)	70 (58, 170)
ALT (IU/L)	35 (25, 58)
NT-proBNP (pg/ml)	952 (520, 3020)
Lactate, (mmol/l)	1.5 (1.1, 2.7)
FiO_2_	0.6 (0.4, 0.8)
PEEP	5 (4, 8)
MAP	7 (5, 13)
Tidal Volume (mL/kg)	8 (6, 10)
OI at inclusion	9 (6, 15)

Abbreviations: WBC, White blood cell; CRP, C-reactive protein; PCT, Procalcitonin; Scr, Serum creatinine; ALT, Alanine aminotransferase; NT-proBNP, N-terminal Pro-Brain Natriuretic Peptide; FiO_2_, Fraction of inspiration oxygen; PEEP, positive end-expiratory pressure; MAP, mean airway pressure; OI, Oxygenation Index

The hospital reference ranges: WBC (10^9^/L): 4.5–9.5; Percentage of neutrophils (%): 45–75, CRP (mg/L): 0–10; PCT (ng/mL): < 0.05, Scr (mmol/L):13–33, ALT (IU/L): 20–40; Lactate, (mmol/l):0.1-1; NT-proBNP (pg/ml): 0-125

The measured data of patients’ physiological indicators in the above table were shown by median (interquartile range)

### Comparison of tNGS and mNGS

tNGS and mNGS were applied to detect pathogens in the BALF samples of all the enrolled children. The total microbial detection rates of tNGS and mNGS were 84.6% (77/91) and 81.3% (74/91), respectively (*P* = 0.55). The top 12 microorganisms detected by tNGS and mNGS are shown in Fig. 1B. The detection results of tNGS and mNGS showed that *Cytomegalovirus* (25 vs. 24), *Acinetobacter baumannii* (15 vs. 15) and *Rhinovirus* (12 vs. 14) ranked top three. No significant difference was found in the distribution of other pathogens, such as bacteria, viruses, and fungi. Among the pathogens detected by tNGS, bacteria, viruses, fungi and other pathogens accounted for 53.4% (86/161), 35.4% (18/161), and 2.5% (4/161) (Fig. 1C). Meanwhile, bacteria, viruses, fungi and other pathogens detected by mNGS accounted for 50.8% (92/181), 35.4% (64/181), 8.3% (15/181) and 5.5% (10/181), respectively (Fig. 1D). As shown in Figs. 1E and 76.9% (70/91) of the patients were positive in both tNGS and mNGS, and 11.0% (10/91) were negative in both detection methods. In addition, 7.7% (7/91) of the patients were positive in tNGS but negative in mNGS (including 2 *Legionella pneumophila*, 2 *Pneumocystis yersinii*, 1 *Enterococcus faecium*, 1 *cytomegalovirus*, 1 *Candida albicans*), and 4.4% (4/91) were positive in mNGS but negative in tNGS (including 2 *Candida parapsilosis*, 1 *Listeria monocytogenes*, 1 *Salmonella typhimurium*). According to the comparison between tNGS and mNGS, the sensitivity, specificity, positive per cent agreement (PPA), and negative per cent agreement (NPA) of tNGS were 95%, 59%, 91%, and 71%, respectively, whereas the sensitivity, specificity, PPA and NPA of mNGS were 91%, 71%, 95%, and 71%, respectively (*P* > 0.05). The overall consistency between tNGS and mNGS was 87.9%, and the kappa value was 0.57 (95% confidence interval, 0.35–0.80) (*P* < 0.001). Compared with tNGS, mNGS also detected 10 other microorganisms, but they were not included in our designed spectrum of 153 respiratory pathogenic microorganisms, including 6 bacteria (3 *Elizabethkingia meningosepticum*, 1 *actinomycetes*, 1 *Salmonella typhimurium*, 1 *Haemophilus parainfluenzae*), 4 fungus (2 *Candida parapsilosis*, 2 *Candida glabrata*), 3 viruses (1 *Anellovirus*, 1 *Polyoma Virus*, 1 *Reoviridae*), and one other pathogen (*Chlamydia Psittacosis*) (Fig. 1F).

In this study, tNGS detected five strains of bacteria carrying drug-resistance genes, including two strains of *S. aureus* with *mec* A gene, one strain of *Klebsiella pneumoniae* with KPC gene, and one strain of *Acinetobacter baumannii* with the OXA gene and one strain of *Escherichia coli* carrying the IMP gene. However, only one strain of *S. aureus* carrying the *mec A* gene was detected in mNGS.

In CMT, the overall microbiological detection rate was 60.2% (55/91) and the three most common pathogens were *Acinetobacter baumannii*, *Klebsiella pneumoniae*, and *Staphylococcus aureus*. In the 10 children who were negative for both tNGS and mNGS, one *Enterococcus faecalis* was detected on CMT and clinically confirmed as the causative agent.

### Treatment and prognosis

In this study, all patients received empiric antibiotic therapy. After clarification of the pathogenesis, we adjusted the anti-infective regimen according to the patient’s clinical characteristics and the results of the pathogen test. Overall, antimicrobial adjustments were made in 55/91 (63%) patients: in 25% of cases, a new antibiotic was added or the current antibiotic was upgraded, while in 38% of cases, the antimicrobial range was narrowed or antimicrobial step-down adjustments were made due to the identification of clinically relevant pathogens. The outcomes of the included patients are shown in Table 3. In this study, all patients received empiric antibiotic therapy. Bronchoscopy was used for diagnosis or treatment and BALF samples were taken before antibiotics were administered.

**Table 3:** Outcomes of the included patients^a^

Variable	Patients (*n* = 91)
In-hospital mortality	2
ECMO, n (%)	1
CRRT, n (%)	5
Duration of invasive ventilation (days; mean ± SD)	2.6 ± 1.1
CICU length of stay (days; mean ± SD)	5.5 ± 2.8
Total hospital length of stay (days; mean ± SD)	15.8 ± 4.0

ECMO, extracorporeal membrane oxygenation; CRRT, continuous renal replacement therapy; CICU, cardiac intensive care unit

^a^Data reported as number and percentage or mean ± standard deviation

## Discussion

Rapid and accurate identification of pathogens is critical for treating pulmonary infections and improving outcomes. At present, the screening of pathogenic bacteria in pulmonary infection mainly relies on traditional methods such as culture methods and automated microbial identification systems. However, these traditional detection methods have shortcomings such as low throughput, long detection time, and the effects of subjective factors on the separation process. Recently, the newly developed mNGS assay, a rapid, high-throughput pathogen detection method, has been applied to BALF samples in several studies [13–17]. Since then, it has become a promising pathogen detection technology. However, owing to the effect of human-derived sequences, mNGS requires a larger amount of data read, a high cost, and a longer detection time (24 h) [18]. Thus, a new approach is needed to address these limitations. The tNGS sample library only needs 0.8 thousand reads, which significantly reduces the sequencing cost and detection time (tNGS and mNGS: $150 vs. $500; 12 h vs. 24 h). Furthermore, tNGS can quantify pathogens at the copy number level, whereas mNGS can only perform relative quantification. Based on the potential advantages of tNGS in pathogen identification, this study is the first to compare the detection efficiency of tNGS and mNGS in infant BALF samples. We found that tNGS and mNGS have comparable sensitivities in detecting pathogens in children’s infected BALF, but tNGS has better timeliness, is economical, and has potential application prospects.

The tNGS technology used in this study targets highly conserved regions of 153 lower respiratory tract pathogens. It designs specific primers and uses multiplex PCR combined with NGS technology for gene sequencing, which are then compared with a gene library to identify pathogens. This method covers at least 95% of common clinical lower respiratory tract pathogens and includes 15 drug-resistance genes, making it suitable for detecting multiple pathogenic microorganism infections. tNGS amplifies and enriches pathogenic nucleic acids using multiplex PCR and removes human gene sequences and other non-target nucleic acid fragments during purification. This compensates for the limitations of metagenomic sequencing, allowing for high-throughput identification of infectious pathogens [8]. Additionally, tNGS enhances the release of nucleic acids from viruses, fungi, and intracellular bacteria through grinding and lysing, improving detection rates. The study indicates that tNGS and mNGS have comparable sensitivities with moderate consistency (kappa = 0.57). However, tNGS offers a shorter detection time (12 vs. 24 h), which can reduce the duration of empirical antibiotic therapy, hospital stays, and improve patient outcomes by initiating appropriate antimicrobial treatments sooner [19]. These benefits translate into better resource utilization and reduced healthcare costs. Early pathogen identification and treatment can lower the risk of complications from delayed or inappropriate therapy, improving the prognosis of infants with severe pneumonia post-congenital heart surgery. Moreover, the cost-effectiveness of tNGS (costing $150 vs. mNGS’s $500) makes it a more viable option for widespread clinical use, particularly in resource-limited settings, alleviating financial burdens and enabling more frequent pathogen detection and monitoring.

Accurate identification of the association of resistance gene profiles with antimicrobial treatment outcomes will help develop individualized treatment plans for children with pulmonary infections. Antimicrobial susceptibility testing (AST) is a traditional method of measuring bacterial antimicrobial resistance. However, AST requires microbiological facilities and trained clinical microbiologists. Furthermore, AST is only effective against culturable bacteria and has limitations in the study of antimicrobial resistance in currently unculturable bacteria and complex microbial communities [20]. Drug resistance in bacteria is often genetically encoded and specific mechanisms include overexpression or duplication of existing genes, point mutations or acquisition of completely new genes through horizontal gene transfer [21]. Improvements in NGS technologies and computational methods facilitate the rapid identification of microbial resistance genes [22]. The number of microbial sequences obtained by mNGS is < 5% of the total sequence of the sample and the specific sequence coverage is < 1% of the microbial genome [23]. Therefore, owing to the effects of mNGS technology, sequencing costs, and databases, the detection of specific drug-resistance genes by conventional mNGS strategies has limitations [24]. However, it is an effective method and its detection efficiency can be improved by performing targeted capture and enrichment of common drug-resistance-related genes for analysis. In our study, tNGS identified five strains carrying drug-resistance genes, compared to one strain detected by mNGS. This capability is crucial for guiding the appropriate use of antibiotics and managing antibiotic resistance. The early detection of resistance genes allows clinicians to tailor antimicrobial therapies more accurately, avoiding ineffective treatments and reducing the spread of resistant pathogens. This not only benefits individual patient care but also has broader public health implications by contributing to the overall effort to combat antimicrobial resistance.

To our knowledge, this study is the first to use tNGS to investigate BALF in infants with pulmonary infection. However, several limitations must be noted. Firstly, the study primarily focused on infants with CHD who underwent surgery, which may limit the generalizability of our findings due to selection bias and the single-hospital, retrospective design. Detection bias is also present as tNGS targets specific pathogens, potentially missing emerging or rare pathogens. Additionally, while tNGS provides information on pathogen-resistance genes, it cannot offer drug-susceptibility results, necessitating empirical adjustments post-results. Data interpretation bias exists since tNGS results rely on established criteria that may not cover all clinical scenarios, and PCR bias during amplification can affect the relative abundance of detected pathogens. Future research should involve larger, multi-center populations and prospective studies to enhance generalizability and eliminate retrospective biases. Expanding the tNGS pathogen panel to include emerging and rare pathogens and developing standardized interpretation guidelines can improve comprehensiveness and reduce subjective biases.Comparing tNGS with mNGS and traditional diagnostic methods will provide a comprehensive evaluation of their strengths and limitations. Conducting detailed cost-benefit analyses will assess the economic feasibility of large-scale tNGS implementation. In cases where tNGS is challenging, mNGS and conventional assays may serve as complementary tools for pathogen detection and clinical management.

## Conclusion

tNGS has comparable sensitivity to mNGS for detecting pathogens in the BALF of children with pulmonary infections, is more timely and economical, and may become a new and more cost-effective method for detecting respiratory pathogens.

## Electronic supplementary material

Below is the link to the electronic supplementary material.

Supplementary Material 1

Supplementary Material 2

Supplementary Material 3

## Data Availability

The datasets used and/or analysed during the current study available from the corresponding author on reasonable request.
