# Supplementary material for: Comparison of targeted next-generation sequencing and metagenomic next-generation sequencing in the identification of pathogens in pneumonia after congenital heart surgery: a comparative diagnostic accuracy study
Source: Ital J Pediatr. 2024 Sep 12;50:174. doi: 10.1186/s13052-024-01749-z (PMC11395185; doi:10.1186/s13052-024-01749-z)
Supplement: Supplementary file 1 — Supplementary Material 1 [file 13052_2024_1749_MOESM1_ESM.docx]

**Supplementary Table-1 153 pathogens detected by tNGS in this study**

| **Primary classification** | **Secondary classification** | **Specific pathogen** |
| --- | --- | --- |
| Bacteria (65 kinds) | Gram-positive cocci | *Staphylococcus aureus, Streptococcus pneumoniae, Streptococcus pyogenes, Streptococcus lactis, Enterococcus faecalis, Enterococcus faecalis* |
|  | Gram-positive bacilli | *Whipple trophoblast, Bacillus cereus, Bacillus anthracis, Corynebacterium pseudotuberculosis, Corynebacterium diphtheriae, occult pyogenes, Rhodococcus equina, Mycobacterium tuberculosis complex, nontuberculous Mycobacterium, Mycobacterium avium, intracellular Mycobacterium, Mycobacterium Kansas, Mycobacterium Chelonii, Mycobacterium abscessum, incidental Mycobacterium, Mycobacterium tuberculosis, Mycobacterium tuberculosis Mycobacterium ulcerans, Mycobacterium Haemophilus, Mycobacterium Gordon, Mycobacterium Bufonis, Mycobacterium vaccae, Mycobacterium Malmo, Nocardia astrosus, Nocardia brasiliensis, Nocardia sunken, and Gelsenkirchnobacteria* |
|  | Gram-negative cocci | *Escherichia coli, enteric salmonella, Klebsiella pneumoniae, Klebsiella acidogenes, Klebsiella aerogenes, Enterobacter cloacae, Serratia marcescens, Proteus mirabilis, Pseudomonas aeruginosa, Burkholderia cepacia, Burkholderia melitensis, Burkholderia melitensis, Stenotrophomonas maltophilia, Acinetobacter baumannii, Moraxella catarrhalis, Elizabethella meningosepticum, Haemophilus influenzae, Haemophilus haemolyticus, Pasteurella multocida, Francisella tularensis, Bordetella pertussis, Bordetella parapertussis, Bordetella avium, Bordetella hodgsonii, Legionella pneumophila, Bacteroides fragilis* |
|  | Rickettsia | *Rickettsia typhi, Rickettsia prussii, Rickettsia Rickettsia, Orientia tsutsugamushi, benakosia* |
| Virus (68 kinds) | DNA virus | *Human Boca virus type 1, human Boca virus type 2, human Boca virus type 3, human Boca virus type 4, human parvovirus B19, human adenovirus, human adenovirus group B, human adenovirus B3, human adenovirus B7, human adenovirus B55, human adenovirus group C, human adenovirus C1, human adenovirus C2, human adenovirus C5, human adenovirus group E, human adenovirus E4, human herpesvirus 1, human herpesvirus 2, human herpesvirus 3, human herpesvirus 4, human herpesvirus 5, human herpesvirus 6, human herpesvirus 7, BK polyomavirus, JC polyomavirus* |
|  | RNA virus | *Influenza A virus, influenza A virus H1N1 2009, influenza A virus H1N1, influenza A virus H3N2, influenza A virus H5N1, influenza A virus H7N9, influenza A virus, influenza C virus, human metapneumovirus, human coronavirus 229E, human coronavirus HKU1, human coronavirus NL63, human coronavirus OC43, mumps virus, human parainfluenza virus 1 Human parainfluenza virus type 2, human parainfluenza virus type 3, human parainfluenza virus type 4, human respiratory syncytial virus type A, human respiratory syncytial virus type B, herpes virus, measles virus, enterovirus, enterovirus group A, Coxsackie virus A2, Coxsackie virus A6, Coxsackie virus A16, enterovirus 71, enterovirus group B, Coxsackie virus A9, Coxsackie virus B2 Coxsackie virus B3, Coxsackie virus B5, Coxsackie virus B6, ecovirus, enterovirus group C, enterovirus group D, enterovirus D68, rhinovirus, rhinovirus A, rhinovirus B, rhinovirus C, rotavirus* |
| Fungus (14 kinds) | Fungus | *Candida albicans, Cryptococcus neoformans, Cryptococcus gattii, histoplasmodium capsulatum, marneffei basket, Aspergillus fumigatus, Rhizopus fimbriae, Rhizopus oryzae, Rhizopus Microsporum, Mucor Rhizopus minimus, sartorium acuminatum, Schizophyllum, hyphomyces asahii, Pneumocystis yersini* |
| Mycoplasma / Chlamydia (6 kinds) | Mycoplasma | *Mycoplasma pneumoniae, Ureaplasma parvum, Ureaplasma urealyticum* |
|  | Chlamydia | *Chlamydia pneumoniae, Chlamydia trachomatis, Chlamydia psittaci* |
