# Supplementary material for: Comparison of targeted next-generation sequencing and metagenomic next-generation sequencing in the identification of pathogens in pneumonia after congenital heart surgery: a comparative diagnostic accuracy study
Source: Ital J Pediatr. 2024 Sep 12;50:174. doi: 10.1186/s13052-024-01749-z (PMC11395185; doi:10.1186/s13052-024-01749-z)
Supplement: Supplementary file 2 — Supplementary Material 2 [file 13052_2024_1749_MOESM2_ESM.docx]

**Supplementary Table-2 15 drug-resistance gene detected by tNGS in this study**

| **Primary classification** | **Secondary classification** | **Specific gene** |
| --- | --- | --- |
| Carbapenem-resistant Enterobacteriaceae (CRE) | Class A ß-lactamases | blaKPC |
|  |  | blaSME |
|  |  | blaIMI |
|  |  | blaGES |
|  | Class B ß-lactamases | blaNDM |
|  |  | blaIMP |
|  |  | blaVIM |
|  |  | blaSPM |
|  |  | blaGIM |
|  | Class D ß-lactamases | blaOXA-48 |
| Methicillin-Resistant Staphylococcus aureus (MRSA) | / | mecA |
| Macrolide-Resistant Mycoplasma pneumoniae (MRMP) | / | A2063G、A2064G、 A2067G、C2617G |
