# Supplementary material for: Comparison of targeted next-generation sequencing and metagenomic next-generation sequencing in the identification of pathogens in pneumonia after congenital heart surgery: a comparative diagnostic accuracy study
Source: Ital J Pediatr. 2024 Sep 12;50:174. doi: 10.1186/s13052-024-01749-z (PMC11395185; doi:10.1186/s13052-024-01749-z)
Supplement: Supplementary file 3 — Supplementary Material 3 [file 13052_2024_1749_MOESM3_ESM.docx]

Dear Editor,

Greetings! I am Dr. Cao from the Department of Cardiac Surgery at Fujian Children’s Hospital, writing to submit our recent research report comparing targeted next-generation sequencing (tNGS) and metagenomic next-generation sequencing (mNGS) for pathogen detection in postoperative pneumonia after congenital heart surgery to your esteemed journal.

Our study found tNGS to have comparable sensitivity and specificity to mNGS, but with faster turnaround times and lower costs. This suggests tNGS could be a promising approach for respiratory pathogen detection.

We believe this research will greatly benefit pediatricians in better understanding and applying tNGS technologies for pathogen detection. The study was rigorously conducted and the results are reliable and compelling. If published, it will provide clinicians new evidence to help select the most appropriate pathogen detection methods to better care for children with congenital heart disease.

We sincerely hope you will consider this work for publication in your journal. Please let me know if you or the reviewers have any questions or suggestions, as we welcome the opportunity to address them and make revisions. Thank you again for your consideration. Wishing you the very best in your work and life!

Sincerely,

Dr. Cao
